# Coregulation: A Multilevel Approach via Biology and Behavior

**DOI:** 10.3390/children10081323

**Published:** 2023-07-31

**Authors:** Marc H. Bornstein, Gianluca Esposito

**Affiliations:** 1 Child and Family Research, Eunice Kennedy Shriver National Institute of Child Health and Human Development, National Institutes of Health, Bethesda, MD 20892, USA; 2 Department of Psychology and Cognitive Science, University of Trento, 38068 Trento, Italy; gianluca.esposito@unitn.it

**Keywords:** coregulation, self-regulation, neural attunement

## Abstract

In this article, we explore the concept of *coregulation*, which encompasses the mutual adaptation between partners in response to one another’s biology and behavior. Coregulation operates at both biological (hormonal and nervous system) and behavioral (affective and cognitive) levels and plays a crucial role in the development of self-regulation. Coregulation extends beyond the actions of individuals in a dyad and involves interactive contributions of both partners. We use as an example parent–child coregulation, which is pervasive and expected, as it emerges from shared genetic relatedness, cohabitation, continuous interaction, and the influence of common factors like culture, which facilitate interpersonal coregulation. We also highlight the emerging field of neural attunement, which investigates the coordination of brain-based neural activities between individuals, particularly in social interactions. Understanding the mechanisms and significance of neural attunement adds a new dimension to our understanding of coregulation and its implications for parent–child relationships and child development.

## 1. Defining Coregulation

Psychologists, psychiatrists, ethologists, and researchers studying interpersonal relationships and human development have used various terms to describe the special nature of well-functioning dyadic relationships, including bidirectionality, coaction, coherence, concordance, contingency, coordination, covariation, harmony, intersubjectivity, matching, mirroring, mutuality, reciprocity, responsiveness, and synchrony. In this article, we use the term coregulation to capture the adaptation of partners in adjusting to each other’s biology and behavior. Coregulation is defined as the continuously unfolding individual attributes and actions that are susceptible to being modified by the changing attributes and actions of a partner [1].

Coregulation operates at both behavioral (affective and cognitive) and biological (hormonal and nervous system) levels and involves the transacting contributions of partners, superseding individual actors in an interaction. Coregulation is dyadic, dynamic, and holistic. Coregulation is believed to have deep roots in evolution and physiology. In this article, we focus on parent–child coregulation for illustration purposes. So, for example, many theorists have argued that parents are biologically predisposed to intuitively attune to their infants, and infants, in turn, are biologically prepared to engage in and expect attuned interactions with parental caregivers [2,3,4,5,6,7].

Coregulation involves bidirectional linkages between partners in recursive patterns [1]. Not all aspects of our abilities to share experiences and exhibit synchronous behaviors in interactions are necessarily conscious. Some coregulation processes unfold slowly or occur quickly without comprehension [8]. Statistical tests of dependence support coregulation, which is based on associations but cannot always unpackage direction of effects between members of a dyad. Concordance [9] represents covariation in partners’ rank-order status, whereas similarity [10] describes equivalent mean levels of specific constructs, structures, functions, or processes between partners. Another perspective on coregulation focuses on contingency, which touches on mutual causality [11].

Importantly, coregulation serves as a critical precursor to self-regulation. Because human infants are highly dependent on caregivers for survival, the emergence of self-regulation primarily occurs within a relational developmental context. The capacity for self-regulation emerges from coregulatory processes between the self and parent–infant interactions [12]. Thus coregulation between parent and child involves the coordination of biological and behavioral systems that support the development of the child’s own regulatory systems. While the literature has primarily focused on the regulation of the child by the parent, both parent and child play essential roles in coregulation. This article explores a multilevel approach to parent–child coregulation, drawing on examples of hormonal, autonomic and central nervous system, and behavioral coregulation between parents and children, ranging from inner processes of hormonal, physiological, and neurological coregulation to manifest levels of behavioral coregulation.

## 2. Hormonal and Sympathetic Nervous System Coregulation

Several hormones are implicated in neuroendocrine processes of parent–child coregulation. A hormone is a signaling molecule that originates from glands in the body. Hormones travel through the circulatory system, reaching distant organs and tissues to regulate a wide range of physiological processes and activities. These include metabolism, reproduction, growth and development, movement, respiration, digestion, tissue function, sensory perception, sleep, excretion, lactation, the stress response, and mood regulation [13]. Two examples of hormones and an enzyme that are sympathetic nervous system biomarkers involved in parent–child coregulation are oxytocin, cortisol, and alpha-amylase.

Oxytocin is a peptide hormone and neuropeptide normally produced by the hypothalamus and released by the pituitary. Oxytocin plays a role in social bonding and childbirth [14]. Higher levels of oxytocin are generally associated with more sensitive and synchronous parental behaviors in mothers and fathers [15]. In a prospective longitudinal study, cohabitating mothers and fathers and their firstborn infants were visited at home during the first postpartum weeks and again after 6 months. Maternal oxytocin was related to maternal-typical affectionate parenting, including infant-directed speech, expressions of positive affect, and loving touch [16,17,18,19]; paternal oxytocin was related to paternal-typical interactions with infants, such as proprioceptive contact, tactile stimulation, and object presentation [20,21]. Notably, maternal oxytocin was unrelated to fathering behaviors, and paternal oxytocin was unrelated to maternal behaviors. A study by Cataldo and colleagues [22] investigated relations among oxytocin receptor gene (OXTr) variations, parental bonding, and prefrontal responses to infants and adults’ cries using near-infrared spectroscopy (NIRS is a non-invasive technique used to measure changes in oxygen levels, and it is commonly employed with infants and children to assess brain activity during various tasks or medical conditions; despite its usefulness, NIRS has limitations, such as shallow penetration depth and susceptibility to motion artifacts, which need to be consid- ered in its interpretation). Cataldo and colleagues [22] found that specific allelic variations of the oxytocin receptor gene polymorphisms regulate physiological modulation of human behavior, especially concerning responses to social cues and affiliative behaviors. The study included 102 young adults who were assessed for OXTr rs53576 and rs2254298 genotypes, recalled parental bonding using the Parental Bonding Instrument—PBI [23], and had their neural responses to social stressors recorded with NIRS. The results revealed that individuals with a higher genetic susceptibility (G/G homozygous) to environmental factors and positive early life interactions exhibited greater promptness to action to general social cues. Furthermore, the dimensions of parental bonding had lateralized effects on prefrontal cortex activation. Greater activation was observed in the right prefrontal cortex for the Care subscale of the PBI, which examines the extent to which affection and sensitive parenting were perceived to be provided by both parents, highlighting the importance of positive caregiving experiences. In contrast, activation in the left prefrontal cortex was associated with overprotection (a dimension of parental bonding characterized by excessive control and overbearing behavior towards the child, also measured using the PBI, suggesting a distinct neural response related to this dimension of parental bonding). This study by Cataldo and colleagues [22] This study provides evidence that genetic variations in the oxytocin receptor gene and the quality of parental bonding contribute to individuals’ neural responses to social cues and stressors. Oxytocin is also released in nursing infants, and oxytocin levels in 4- to 6-month-olds increase after interactions with caregivers, with higher levels related to greater parent–child affective synchrony and social engagement [24]. Oxytocin coregulation illustrates one type of hormonal attunement between parent and infant.

Cortisol, a steroid hormone synthesized by the adrenal gland, is released in response to stress. Its primary functions include raising blood sugar levels, suppressing the immune system, and facilitating the metabolism of fat, protein, and carbohydrates [25]. Individual differences in cortisol levels are associated with psychological stress, arousal, and negative emotionality [26]. Cortisol levels rise during pregnancy to increase vigilance. Higher cortisol levels on postpartum days 3 and 4 are associated with maternal approach behaviors and positive attitudes [27,28]. Nearly 40% of mothers’ cortisol crosses the placenta [29]. Gitau et al. [30] measured plasma cortisol concentrations in paired maternal and fetal venous samples at 13 to 35 weeks of gestation. Maternal cortisol levels were higher compared to fetal levels, with a maternal-to-fetal ratio of 11:4. Notably, fetal cortisol concentrations were aligned with maternal cortisol concentrations, indicating concordance. Furthermore, maternal and infant cortisol levels exhibited synchronicity [31,32,33,34]. Associations in salivary cortisol levels are evident between mothers and their infants, preschoolers, elementary school-aged children, as well as adolescent offspring [32,34,35,36]. Notably, the associations observed in salivary cortisol levels extend beyond genetically related individuals, encompassing unrelated individuals such as young adults involved in dating relationships and spouses in long-standing marriages [37,38]. Cortisol and testosterone have been extensively analyzed in relation to various aspects of human physiology and behavior, including their potential roles in parenting behavior. The interplay between these two hormones has been of particular interest in understanding the complexities of caregiving dynamics between parents and their children. In a study conducted by Bos and colleagues [39], the objective was to examine the connection between the observed quality of caregiving during parent–child interactions and the pre- and postnatal cortisol and testosterone levels in both mothers and fathers. The sample for this study included 88 mothers and 57 fathers who engaged in parent–child interactions. To evaluate basal levels and steroid reactivity, cortisol and testosterone were assessed before and after interactions with an infant simulator (during the prenatal period) and with their own child (during the postnatal period). The researchers postulated that the combination of cortisol and testosterone levels would be linked to the quality of caregiving displayed by parents. The findings revealed notable differences between mothers and fathers in terms of the associations between the two hormones and caregiving quality. In fathers, the interactions between cortisol and testosterone played a crucial role in predicting caregiving quality both before and after the birth of their child. Specifically, fathers with lower cortisol levels showed a stronger negative relation between testosterone and caregiving quality during the prenatal period. This result suggests that the interplay between cortisol and testosterone levels may influence how fathers engage in caregiving behaviors even before the birth of their child. Furthermore, during the postnatal testing, fathers with higher testosterone levels exhibited a stronger negative association between cortisol and caregiving quality. These findings suggest that higher levels of testosterone in fathers, combined with lower cortisol levels, may have implications for their caregiving behaviors. This study also found that prenatal cortisol levels were related to paternal caregiving quality during interactions with their child, further emphasizing the potential influence of hormonal factors on paternal responsiveness. In contrast, no significant associations were observed between caregiving quality and the endocrine measures in mothers. This lack of association between cortisol, testosterone, and caregiving quality in mothers warrants further investigation and may suggest that other factors, such as oxytocin or progesterone, could play more instrumental roles in maternal caregiving behaviors. Overall, the study by Bos and colleagues provides valuable insights into the intricate in- terplay between cortisol and testosterone in shaping parenting behaviors, particularly in fathers. These findings contribute to the growing body of research exploring the hormonal antecedents of human caregiving behavior and highlight potential sex differences in the hormonal regulation of parental involvement and responsiveness.

Salivary alpha-amylase is an enzyme biomarker for sympathetic nervous system activity. Davis and Granger [40] established that maternal and infant salivary alpha-amylase levels are positively correlated at 6, 12, and 24 months of age, providing evidence for synchrony in maternal and infant sympathetic nervous system coregulation. Levels were not associated at 2 months, suggesting that sympathetic coregulation of salivary alpha-amylase matures or depends on shared experience. It is important to note at this juncture that many studies in the tradition of documenting coregulation are correlational in nature, and establishing the direction of effects in a correlation remains a challenge. As the truism goes, “Correlation is not causation”. In consequence, further research is needed to fully elucidate complex hormonal processes underlying caregiving behaviors in mothers and fathers alike as well as their implications for child development.

## 3. Autonomic Nervous System Coregulation

The autonomic nervous system (ANS) controls the involuntary and primarily subconscious physiology-regulating functions through up-regulation (arousing) and down-regulation (soothing). Cardiac function and blood pressure are important components of the ANS, which assess self-regulatory physiological processes that function to maintain internal homeostasis. Children and mothers share characteristic autonomic response styles that are reflected in similar patterns of ANS co-regulation. Indeed, parent–offspring correlations in such rapidly fluctuating ANS indices as heart rate and blood pressure have been reported [41], even when parents and children are tested on different occasions using different procedures. A hypothesized function of ANS coregulation for the child may be to facilitate biological and behavioral homeostatic self-regulation.

Cardiac vagal tone is defined as the level of activity in the vagus nerve that influences heart rate and is conceptualized as an index of stress [42]. It has been applied to understand physiological substrates of self-regulation, information processing, temperament, and emotion from infancy through adulthood [43]. Vagal regulation during environmental challenge—the capacity to engage and disengage vagal outflow—is an appropriate response to stimulation or stress. Baseline-to-task change in vagal regulation serves as an index of vagal regulatory function. Bornstein and Seuss [44] measured baseline and task vagal regulation in mothers and their children at 2 months and at 5 years and calculated parent–child coregulation at baseline and as baseline-to-task change. Although no baseline coregulation was found, baseline-to-task change in vagal regulation showed marginally significant mother–child concordance at 2 months and significant coregulation at 5 years. Vagal tone in depressed mothers and their infants resemble one another as well [45], an association that could reflect coregulation or similar mother and child styles of approach to tasks.

Several studies have specifically focused on how the autonomic nervous system of infants responds to maternal stimuli, highlighting the bidirectional influence between mothers and their infants. Esposito and colleagues [46] examined infant calming responses during maternal carrying in both humans and mice. This study aimed to investigate the physiological and behavioral effects of maternal carrying on infants and to explore processes involved in this response. The authors [46] reported experiments on human infants and mouse pups, observing similar calming responses in both species when carried by their mothers. These responses included decreasing of distress vocalizations, reduced motor activity, and increased heart rate variability (HRV), indicating a more adaptive autonomic state. Furthermore, Ref. [46] explored the neural pathways involved in the calming response to maternal carrying. They identified the involvement of the parasympathetic nervous system, particularly the vagus nerve, in regulating heart rate variability during maternal carrying. Activation of the vagus nerve was associated with observed increase in HRV, indicating enhanced autonomic regulation.

Another relevant study in this area is a recent work by [47], which employed a combination of physiological analyses and dynamic mother–infant interactions to disentangle the intricate responses of infants to maternal holding and transport. The findings demonstrated that infants’ cries diminished when their mothers carried them or when reciprocal motion was facilitated by a moving cot, highlighting the significance of both maternal carrying and motion in soothing infants. Maternal holding alone did not exhibit the same effect. The authors also found that 5-min carrying promoted sleep in crying infants, even during the daytime when infants were typically awake.The study also revealed that the sleep outcome after laydown was associated with the sleep duration before the laydown onset. Together, these studies [46,47] provide valuable insights into the automatic responses of the infant autonomic nervous system to maternal stimuli. They shed light on the physiological and neurobiological mechanisms underlying the calming effects of maternal carrying, involving factors such as oxytocin signaling, vagal activation, and enhanced autonomic regulation. Understanding these processes can contribute to the development of effective interventions for soothing and promoting sleep in infants, ultimately benefiting both infants and their parents. There is a long tradition in the field of comparative physiology surrounding human and mice carrying and calming behaviors. Nonetheless, this literature should be supported with additional research to determine if these behaviors are synonymous across species. Drawing direct inferences about human behavior from mouse studies may not be entirely appropriate without further investigation and validation. More research is needed to enhance our understanding of these behaviors and their potential cross-species similarities and implications.

Several studies have also investigated autonomic nervous system responses of mothers to their infants’ stimuli, highlighting the intricate and bidirectional nature of the mother–infant relationship. Two notable examples of such studies are the research conducted by Ohmura and colleagues [48] and by Doi and colleagues [49]. Ohmura and colleagues [48] explored maternal physiological calming responses during breastfeeding. They investigated maternal activities and autonomic nervous system dynamics using behavioral measures and a Holter electrocardiogram. The study revealed that during breastfeeding, mothers exhibited reduced verbal communication and lower heart rate compared to sitting with the infant without breastfeeding. Moreover, measurements of maternal heart rate variability indicated higher parasympathetic activity during breastfeeding. These findings suggested that somatosensory stimuli of breastfeeding, such as tactile stimulation at the breast, activate parasympathetic activity in mothers, facilitating a calming response in the infants. Doi and colleagues [49] focused on the inaudible components of the human infant cry and their influence on maternal hemodynamic responses. The researchers found that the human infant cry contains ultrasonic components, similar to distress vocalizations in other mammalian species. Notably, mothers themselves were not consciously aware of these ultrasonic components, but their presence, in combination with audible components, led to increases in oxygenated hemoglobin concentrations in mothers’ breast region. This modulation occurred when the body surface was exposed to the ultrasonic components, providing novel evidence of the role played by ultrasonic signals in human mother–infant interaction. These studies exemplify autonomic nervous system responses of mothers to infant stimuli. The maternal calming responses observed during breastfeeding and the modulation of hemodynamic responses by ultrasonic components of the infant cry underscore the complex and dynamic nature of maternal–infant interactions. Understanding these automatic physiological responses can contribute to a deeper comprehension of processes underlying the abiding and unique bond between mothers and their infants, ultimately benefiting both.

## 4. Central Nervous System Coregulation

The central nervous system (CNS), consisting of the brain and spinal cord, integrates information it receives from, and coordinates and influences the activity of, all parts of the body. Some CNS areas (see below) have been described as the mirror neuron system and support spontaneous imitation and intersubjectivity between mothers and infants at the level of the CNS [50,51,52,53,54]. Imitation and intersubjectivity are processes that give evidence of coregulation.

Presenting mothers with smiling pictures of their own infants compared to unfamiliar infants leads to heightened brain activity [55,56]. Furthermore, the brain activation of mothers viewing pictures of their own infants is positively correlated with pleasant mood ratings and affective responses towards their child [57]. In a study conducted by Strathearn and colleagues [56], functional magnetic resonance imaging (fMRI) was employed to examine the neural responses of mothers to their own infant’s facial expressions, specifically comparing happy, neutral, and sad faces. The research involved 28 first-time mothers who were presented with novel face images of their own 5- to 10-month-old infant as well as a matched unknown infant. The findings revealed that key brain regions associated with dopamine-mediated reward processing exhibited activation when mothers viewed their own infant’s face compared to an unfamiliar infant’s face. These regions included the ventral tegmental area/substantia nigra, the striatum, and various frontal lobe areas involved in emotion processing, cognition, and motor/behavioral outputs. Importantly, happy infant faces specifically activated interconnected regions mediated by dopaminergic neurons, such as the substantia nigra and dorsal putamen. The activation in these regions was associated with positive infant affect, with happy faces eliciting stronger responses compared to neutral and sad faces. This study provides valuable insights into neural mechanisms that underlie the distinctive bond between mothers and their infants, offering a glimpse into the neural basis of mother-infant attachment and the integration of affective and cognitive information in maternal caregiving.

Bornstein and colleagues Bornstein et al. [58], Esposito and colleagues [59], and Mash and colleagues [60] took pictures of mothers and babies visiting their laboratory and showed pictures of their own and appearance-matched babies to mothers, and pictures of their own and appearance-matched mothers to babies, for 500 ms each while recording EEG. Differentiated electrical responses of 3-month-old infants when presented with images of familiar and unfamiliar faces were observed in three specific time windows: 370–480 ms, 610–690 ms, and 830–960 ms. These responses demonstrated event-related synchronization or desynchronization in the beta or gamma frequency bands at specific sites including the left frontal, midline central, bilateral temporal, and right parietal regions. These findings provide evidence of organized brain activity underlying maternal face recognition in very young infants. Additionally, the study revealed that the maternal nervous system becomes attuned through just 3 months of experience with their own infant. When primipara mothers of the 3-month-olds viewed images of their own infant and an unfamiliar but appearance-matched infant, distinctive late-wave responses (N/P600 “familiar/novel”) were observed, indicating recognition sensitivity based on their 3 months’ experience with their own infant’s face. These findings highlight the specialized nature of infant brain responses to their mother at an early age, suggesting that information in the mother–child dyad is processed differently as a result of co-regulation.

Human infant faces also trigger a network of brain activation in mothers involving premotor regions and the supplementary motor area (SMA), which are implicated in preparation and intention to move and respond and to communicate [61,62,63]. SMA, along with lateral premotor areas, generates a “readiness potential” that antecedes movement and is considered the neural correlate of intentional movement planning that can be measured even when people are unaware of their intention to move [64,65,66,67]. Neuroimaging studies indicate that infant faces activate a “readiness” to interact with babies. SMA (called the “starting mechanism of speech” [68,69]) is also critical in preparing a verbal utterance and initiating vocal tract movements during speech production. In an fMRI study, Caria et al. [70] found enhanced activity of SMA to human infant faces vis-à-vis faces of infant animals and mature humans and animals. Caria also found enhanced brain activation patterns commonly associated with emotion recognition and evaluation [71,72], as well as simulation of others’ emotional experiences [73,74]. Activation of the thalamo-cingulate circuit and insula occurs when participants decode another person’s emotional states based on facial cues and then evaluate their own emotional responses to those faces [54,75]. This observed neural activity may subserve adults’ readiness to empathize with infants’ emotional expressions, a vital ingredient of coregulation.

## 5. Neural Coregulation

The concept of neural attunement has emerged as an innovative approach in neuroscience, directing attention to the investigation of human sociability within structured or ecologically valid real-time reciprocal social interactions [76,77,78,79,80]. Traditional cognitive studies often emphasize individual processes; however, there is growing evidence that neural activities can become coordinated between two individuals through environmental signals, such as face-to-face social interactions [81,82,83,84]. This phenomenon, referred to as neural synchrony (or here coregulation), is believed to facilitate effective communication and behavioral coordination between individuals [85,86]. Hyperscanning studies, which involve simultaneous recording of brain activities from multiple individuals, have proven valuable in unraveling neural coregulation in interpersonal dynamics [87]. Through examination of inter-brain correlations, these studies have provided insights into neural mechanisms underlying social interactions [88,89,90]. Notably, cooperative activities are associated with increased neural coregulation or synchrony between brains [83,91,92]. In the context of joint play, for example, theta neural oscillations observed in mothers’ brains predict attention in their 12-month-old infants [93]. However, studies have reported mixed findings regarding the patterns of brain synchronization based on the gender composition of dyads, with some studies demonstrating synchrony differences between same-sex and mixed-sex dyads [94,95].

The nature of the relationship between individuals also plays a role in neural synchronization. For instance, female–male romantic partner dyads have exhibited higher inter-brain synchronization in the right superior frontal cortex compared to other types of dyads, such as female–male friends or strangers [96]. In parent–child dyads engaged in cooperation tasks, neural synchronization has been observed in the bilateral prefrontal cortex and temporo-parietal regions [97]. Moreover, the gender composition of the dyads can influence the patterns of synchronization, as mother–son dyads have shown distinct synchronization patterns when compared to mother-daughter dyads [98].

The quality of social interactions in parent–child dyads is also reflected in neural coregulation. For instance, the attitude of fathers toward their parental role is positively associated with neural synchronization between fathers’ and children’s brains during cooperation, which, in turn, is linked to reduced child psychopathology [85,99]. Emotional quality and tone during interactions, as well as parental stress, also affect neural coregulation in parent–child dyads [100,101,102,103]. The synchronization of neural activities between parents and children sometimes reflects emotional connection in the dyad and contributes to the child’s development of adaptive emotion regulation strategies [104,105].

In summary, neural coregulation refers to the alignment of neural activity between individuals, particularly in the context of social interactions. Neural coregulation is influenced by factors such as the type of relationship, gender composition of dyads, and emotional dynamics. Significant progress has been made in understanding neural coregulation in a short amount of time, but further research is needed to explore the nuanced ways in which such synchrony emerges and is dynamically modulated by different factors at different levels of analysis.

## 6. Behavioral Coregulation

As the foregoing shows, coregulation is a multi-level phenomenon, taking place at hormonal and sympathetic nervous system, autonomic nervous system, and central nervous system levels as well as at brain-to-brain levels. Coregulation is also a regular attribute of interpersonal behavior.

Parents and their offspring share certain psychological characteristics. They exhibit similarities in engaging in physical activity [5,106], cognitive functions [41,107,108,109,110], and even food preferences and disgust or “contamination sensitivity” [111,112]. In addition, mutual parent–child contingencies of eye gaze, facial expressions, prosody in speech rhythms, and attention have been documented [113].

To investigate mother–infant behavioral coregulation, Bornstein and colleagues [114] analyzed vocal contingency data from 796 mother–infant dyads in cultural groups in 11 countries. Mothers vocalize contingently in response to their infants’ vocalizations, and infants tend to vocalize contingently in response to their mothers. Their index of contingency was an odds ratio, the probability that a mother will talk to her infant given that her infant has just stopped vocalizing to her in the last 2 s divided by the mother failing to talk to her infant given that her infant just stopped vocalizing *over* the mother having talked to her infant in the absence of her infant having just vocalized divided by the mother’s not talking given her infant did not vocalize. Maternal vocalization to infants was contingent on infant vocalization in 9 of 11 countries. Five-month infants’ nondistress vocalizations were also contingent on their mothers’ speech to them in approximately one-half of the countries. Moreover, mother and infant contingency scores were related: Mothers who were relatively more responsive to their infants’ vocalizations had infants who were relatively more responsive to their mothers’ vocalizations overall and in 9 of 11 cultural groups. These findings point to the origins of mother–infant vocalization transactions, and vocal turn-taking reinforces a culture-general result about mother–infant vocal coregulation. In a sense, they reflect a requirement of the nervous system: The human nervous system has considerable difficulty processing two sources of vocal information at the same time.

To investigate more general mother–infant behavioral coregulation and do so on a global level as well, Bornstein and colleagues [115] analyzed data from the same cross-cultural data set, and Bornstein [116] analyzed additional data from mother–infant dyads in two cultural groups in each of five countries. Across cultures, mothers and their 5-month infants show noteworthy behavioral coregulation as well as specificity: Mothers who encourage their infants’ physical development more have more physically developed infants; mothers who engage their infants socially more have infants who reciprocate their social attention more; mothers who encourage their infants didactically more have infants who explore properties, objects, and events in the environment more, as do infants whose mothers outfit their environments in a richer way. In summary, mothers and young infants in a variety of ethnicities, socioeconomic statuses, and cultures around the world are behaviorally attuned with one another; moreover, as noted, behavior coregulation tends to be domain specific. It is noteworthy that mother–child behavioral coregulation is also robust and appears at least partially refractory to certain dysfunctions. For example, the total percentage of time spent in matching behavior states is reduced in depressed relative to nondepressed mother–infant dyads, yet cross-spectral analyses of mother and infant behavior-state time series reveal behavioral coherence in depressed and nondepressed dyads alike [117]. Mothers’ emotional relationships with their children with Down syndrome and cancer are equally attuned as those to mothers with typically developing children [118,119,120].

## 7. Conclusions

Parent–child coregulation is pervasive and expectable. There are reasons at many levels—genetic to experiential—for these two parties to coregulate. Moreover, socialization is bidirectional, with parents and children actively assuming reciprocal mutually influential and attuned roles. The two partners co-create their shared history over time, and the two shape their evolving relationship [121,122]. Coregulation between parent and child is a cornerstone of children’s biological, socioemotional, and cognitive well-being and adaptation throughout the balance of the life course [15,123,124,125,126]. Understanding different facets of the behavior and biology of coregulation is a dawning focus of theory and research in the developmental and parenting sciences.

## 8. Future Directions

The studies referenced in this short review form the kernel of a much more thoroughgoing research agendum on parent–child coregulation at multiple levels of life and living. As our understanding of parent–child coregulation continues to evolve, advances in measurement techniques offer exciting possibilities for gaining unprecedented insights into this complex process. Techniques such as hyperscanning, which involves simultaneous neuroimaging of parents and children, have the potential to provide a more comprehensive understanding of the neural underpinnings of coregulation. Hyperscanning techniques, including EEG, fNIRS, and even fMRI, allow researchers to investigate the real-time neural dynamics of parent–child interactions, shedding light on intricate patterns of brain activity that contribute to mutual regulation. By employing these advanced measurement techniques, researchers can explore the neural synchrony, neural activation patterns, and connectivity between parent and child during different interactive tasks. This cutting-edge approach has the potential to reveal neural mechanisms that facilitate successful coregulation and identify potential disruptions in parent–child dyad relationships. Furthermore, hyperscanning techniques can help elucidate how the quality of coregulation relates to various aspects of child development, including socioemotional, cognitive, and behavioral outcomes. While the application of hyperscanning techniques in the study of parent–child coregulation is still in its nascent phase, its promising potential offers a rewarding pathway for future research. With further advances in technology, we anticipate that these measurement techniques will become more accessible, allowing for larger-scale studies and the examination of coregulation in diverse populations and contexts. The field of parent–child coregulation is expanding, with recognition of its significance for child development and parenting science growing. Future research in this area, leveraging innovative measurement approaches, will undoubtedly enhance our understanding of the intricate coregulation of parents and children and its implications for child development and well-being.

## Data Availability

Not applicable.

## References

[1] Butler E.A., Randall A.K. (2013). Emotional Coregulation in Close Relationships. Emot. Rev..

[2] Brazelton T.B., Kobayashi N., Brazelton T.B. (1984). Four early stages in the development of mother–infant interaction. The Growing Child in Family and Society: An Interdisciplinary Study in Parent–Infant Bonding.

[3] Emde R.N., Call J.D., Galenson E., Tyson R.L. (1984). The affective self: Continuities and transformations from infancy. Frontiers of Infant Psychiatry.

[4] Papoušek H., Papoušek M. (2002). Intuitive parenting. Handbook of Parenting Vol. 2 Biology and Ecology of Parenting.

[5] Stern D.N. (1985). The Interpersonal World of the Infant.

[6] Bornstein M.H., Arterberry M.E., Mash C. (2004). Long term memory for an emotional interpersonal interaction occurring at 5 months of age. Infancy.

[7] Tronick E.Z., Adamson L.B., Als H., Brazelton T.B. Infant emotions in normal and pertubated interactions. Proceedings of the Biennial Meeting of the Society for Research in Child Development.

[8] Ham J., Tronick E. (2009). Relational psychophysiology: Lessons from mother–infant physiology research on dyadically expanded states of consciousness. Psychother. Res..

[9] Lee T.H., Qu Y., Telzer E.H. (2018). Dyadic neural similarity during stress in mother–child dyads. J. Res. Adolesc..

[10] Peterson C., Roberts C. (2003). Like mother, like daughter: Similarities in narrative style. Dev. Psychol..

[11] Beebe B., Jaffe J., Markese S., Buck K., Chen H., Cohen P., Bahrick L., Andrews H., Feldstein S. (2010). The origins of 12-month attachment: A microanalysis of 4-month mother-infant interaction. Attach. Hum. Dev..

[12] Hofer M.A. (2006). Psychobiological roots of early attachment. Curr. Dir. Psychol. Sci..

[13] Neave N. (2008). Hormones and Behaviour: A Psychological Approach.

[14] Yang H.P., Wang L., Han L., Wang S.C. (2013). Nonsocial functions of hypothalamic oxytocin. ISRN Neurosci..

[15] Feldman R. (2007). Mother–infant synchrony and the development of moral orientation in childhood and adolescence: Direct and indirect mechanisms of developmental continuity. Am. J. Orthopsychiatry.

[16] Feldman R. (2007). Parent–infant synchrony and the construction of shared timing; physiological precursors, developmental outcomes, and risk conditions. J. Child Psychol. Psychiatry.

[17] Feldman R., Weller A., Zagoory-Sharon O., Levine A. (2007). Evidence for a neuroendocrinological foundation of human affiliation: Plasma oxytocin levels across pregnancy and the postpartum period predict mother–infant bonding. Psychol. Sci..

[18] Feldman R., Eidelman A.I. (2003). Direct and indirect effects of breast milk on the neurobehavioral and cognitive development of premature infants. Dev. Psychobiol..

[19] Feldman R., Eidelman A.I., Rotenberg N. (2004). Parenting stress, infant emotion regulation, maternal sensitivity, and the cognitive development of triplets: A model for parent and child influences in a unique ecology. Child Dev..

[20] Feldman R. (2003). Infant-mother and infant-father synchrony: The coregulation of positive arousal. Infant Ment. Health J..

[21] Parke R.D. (2002). Fathers and families. Handbook of Parenting Vol. 3: Status and Social Conditions of Parenting.

[22] Cataldo I., Neoh M., Chew W., Foo J., Lepri B., Esposito G. (2020). Oxytocin receptor gene and parental bonding modulate prefrontal responses to cries: A NIRS Study. Sci. Rep..

[23] Parker G., Tupling H., Brown L.B. (1979). A parental bonding instrument. Br. J. Med. Psychol..

[24] Feldman R., Gordon I., Zagoory-Sharon O. (2010). The cross-generation transmission of oxytocin in humans. Horm. Behav..

[25] Hoehn K., Marieb E.N. (2010). Human Anatomy & Physiology.

[26] Kirschbaum C., Hellhammer D.H. (1994). Salivary cortisol in psychoneuroendocrine research: Recent developments and applications. Psychneuroendocrinology.

[27] Corter C., Fleming A.S. (1990). Maternal responsiveness in humans: Emotional, cognitive and biological factors. Adv. Study Behav..

[28] Fleming A.S., Steiner M., Anderson V. (1987). Hormonal and attitudinal correlates of maternal behavior during the early postparpregnancy. J. Reprod. Infant Psychol..

[29] Glover V., Teixeira J., Gitau R., Fisk N.M. (1999). Mechanisms by which maternal mood in pregnancy may affect the fetus. Contemp. Rev. Obstet. Gynecol..

[30] Gitau R., Cameron A., Fisk N.M., Glover V. (1998). Fetal exposure to maternal cortisol. Lancet.

[31] Spangler G. (1991). The emergence of adrenocortical circadian function in newborns and infants and its relationship to sleep, feeding, and maternal adrenocortical activity. Early Hum. Dev..

[32] Sethre-Hofstad L., Stansbury K., Rice M.A. (2002). Attunement of maternal and child adrenocortical response to child challenge. Psychoneuroendocrinology.

[33] Stenius F., Theorell T., Lilja G., Scheynius A., Alm J., Lindblad F. (2008). Comparisons between salivary cortisol levels in six-months-olds and their parents. Psychoneuroendocrinology.

[34] Thompson L.A., Trevathan W.R. (2008). Cortisol reactivity, maternal sensitivity, and learning in 3-month-old infants. Infant Behav. Dev..

[35] Kivlighan K.T., Granger D.A., Booth A. (2005). Gender differences in testosterone and cortisol response to competition. Psychoneuroendocrinology.

[36] van Bakel H.J., Riksen-Walraven J.M. (2008). Adrenocortical and behavioral attunement in parents with 1-year-old infants. Dev. Psychobiol..

[37] Brandtstädter J., Baltes-Götz B., Kirschbaum C., Hellhammer D. (1991). Developmental and personality correlates of adrenocortical activity as indexed by salivary cortisol: Observations in the age range of 35 to 65 years. J. Psychosom. Res..

[38] Powers S.I., Pietromonaco P.R., Gunlicks M., Sayer A. (2006). Dating couples’ attachment styles and patterns of cortisol reactivity and recovery in response to a relationship conflict. J. Personal. Soc. Psychol..

[39] Bos P., Hechler C., Beijers R., Shinohara K., Esposito G., de Weerth C. (2018). Prenatal and postnatal cortisol and testosterone are related to parental caregiving quality in fathers, but not in mothers. Psychoneuroendocrinology.

[40] Davis E.P., Granger D.A. (2009). Developmental differences in infant salivary alpha-amylase and cortisol responses to stress. Psychoneuroendocrinology.

[41] Ditto B., France C., Miller S. (1989). Spouse and parent-offspring similarities in cardiovascular response to mental arithmetic and isometric hand-grip. Health Psychol..

[42] Porges S.W. (1995). Cardiac vagal tone: A physiological index of stress. Neurosci. Behav. Rev..

[43] Porges S.W. (2011). The Polyvagal Theory: Neurophysiological Foundations of Emotions, Attachment, Communication, and Self-Regulation.

[44] Bornstein M.H., Seuss P.E. (2000). Child and mother cardiac vagal tone: Continuity, stability, and concordance across the first 5 years. Dev. Psychol..

[45] Jones N.A., Field T., Fox N.A., Lundy B., Hart S. (1998). Newborns of mothers with depressive symptoms are physiologically less developed. Infant Behav. Dev..

[46] Esposito G., Yoshida S., Ohnishi R., Tsuneoka Y., Rostagno M.d.C., Yokota S., Okabe S., Kamiya K., Hoshino M., Shimizu M. (2013). Infant calming responses during maternal carrying in humans and mice. Curr. Biol..

[47] Ohmura N., Okuma L., Truzzi A., Shinozuka K., Saito A., Yokota S., Bizzego A., Miyazawa E., Shimizu M., Esposito G. (2022). A method to soothe and promote sleep in crying infants utilizing the transport response. Curr. Biol..

[48] Ohmura N., Okuma L., Truzzi A., Esposito G., Kuroda K. (2023). Maternal physiological calming responses to infant suckling at the breast. J. Physiol. Sci..

[49] Doi H., Sulpizio S., Esposito G., Katou M., Nishina E., Iriguchi M., Honda M., Oohashi T., Bornstein M.H., Shinohara K. (2019). Inaudible components of the human infant cry influence haemodynamic responses in the breast region of mothers. J. Physiol. Sci..

[50] Gallese V., Rochat M., Legerstee M., Haley D., Bornstein M.H. (2012). The evolution of motor cognition: Its role in the development of social cognition and implications for the Autistic Spectrum Disorder. The Developing Infant Mind: Integrating Biology and Experience.

[51] Killner J.K., Neal A., Weiskopf N., Friston K.J., Frith C.D. (2009). Evidence of mirror neurons in human inferior frontal gyrus. J. Neurosci..

[52] Mukamel R., Ekstrom A.D., Kaplan J., Iacoboni M., Fried I. (2010). Single-neuron responses in humans during execution and observation of action. Curr. Biol..

[53] Rizzolatti G., Sinigaglia C. (2010). The functional role of the parieto-frontal mirror circuit: Interpretations and misinterpretations. Nat. Rev. Neurosci..

[54] Schulte-Rüther M., Markowitsch H.J., Fink G.R., Piefke M. (2007). Mirror neuron and theory of mind mechanisms involved in face-to-face interactions: A functional magnetic resonance imaging approach to empathy. J. Cogn. Neurosci..

[55] Rigo P., Kim P., Esposito G., Putnick D.L., Venuti P., Bornstein M.H. (2019). Specific maternal brain responses to their own child’s face: An fMRI meta-analysis. Dev. Rev..

[56] Strathearn L., Li J., Fonagy P., Montague P.R. (2008). What’s in a smile? Maternal brain responses to infant facial cues. Pediatrics.

[57] Nitschke J., Nelson E., Rusch B., Fox A.S., Oakes T., Davidson R. (2004). Orbifrontal cortex tracks positive mood in mothers viewing pictures of their newborn infants. NeuroImage.

[58] Bornstein M.H., Arterberry M.E., Mash C. (2013). Differentiated brain activity in response to faces of “own” versus “unfamiliar” babies in primipara mothers: An electrophysiological study. Dev. Neuropsychol..

[59] Esposito G., Valenzi S., Islam T., Mash C., Bornstein M.H. (2015). Immediate and selective maternal brain responses to own infant faces. Behav. Brain Res..

[60] Mash C., Bornstein M.H., Arterberry M.E. (2013). Brain dynamics in young infants’ recognition of faces: EEG oscillatory activity in response to mother and stranger. NeuroReport.

[61] Alario F.X., Chainay H., Lehericy S., Cohen L. (2006). The role of the supplementary motor area (SMA) in word production. Brain Res..

[62] Brendel B., Hertrich I., Erb M., Lindner A., Riecker A., Grodd W., Ackermann H. (2010). The contribution of mesiofrontal cortex (SMA) to the preparation and execution of repetitive syllable productions: An fMRI study. NeuroImage.

[63] Riecker A., Mathiak K., Wildgruber D., Erb M., Hertrich I., Grodd W., Ackermann H. (2005). fMRI reveals two distinct cerebral networks subserving speech motor control. Neurology.

[64] Deecke L., Kornhuber H.H. (1978). An electrical sign of participation of the mesial “supplementary” motor cortex in human voluntary finger movement. Brain Res..

[65] Goldberg G. (1985). Supplementary motor area structure and function—Review and hypotheses. Behav. Brain Sci..

[66] Haggard P., Eimer M. (1999). On the relation between brain potentials and the awareness of voluntary movements. Exp. Brain Res..

[67] Jahanshahi M., Jenkins I.H., Brown R.G., Marsden C.D., Passingham R.E., Brooks D.J. (1995). Self-initiated versus externally triggered movements. I. An investigation using measurement of regional cerebral blood flow with PET and movement-related potentials in normal and Parkinson’s disease subjects. Brain.

[68] Ackermann H., Ziegler W., Hardcastle W.J., Laver J., Gibbon F. (2010). Brain mechanisms underlying speech motor control. The Handbook of Phonetic Sciences.

[69] Botez M.I., Barbeau A. (1971). Role of subcortical structures and particularly of the thalamus, in the mechanisms of speech and language. Int. J. Neurol..

[70] Caria A., de Falco S., Venuti P., Lee S., Esposito G., Rigo P., Bornstein M.H. (2012). Species-specific response to human infant faces in the premotor cortex. NeuroImage.

[71] Carr L., Iacoboni M., Dubeau M.C., Mazziotta J.C., Lenzi G.L. (2003). Neural mechanism of empathy in humans: A relay from neural system for imitation to limbic areas. Proc. Natl. Acad. Sci. USA.

[72] Seitz R.J., Schafer R., Scherfeld D., Friederichs S., Popp K., Wittsack H.J., Franz M. (2008). Valuating other people’s emotional face expression: A combined functional magnetic resonance imaging and electroencephalography study. Neuroscience.

[73] Singer T., Seymour B., O’Doherty J., Kaube H., Dolan R.J., Frith C.D. (2004). Empathy for pain involves the affective but not sensory components of pain. Science.

[74] Wicker B., Keysers C., Plailly J., Royet J.P., Gallese V., Rizzolatti G. (2003). Both of us disgusted in my insula: The common neural basis of seeing and feeling disgust. Neuron.

[75] Shamay-Tsoory S.G., Aharon-Peretz J., Perry D. (2009). Two systems for empathy: A double dissociation between emotional and cognitive empathy in inferior frontal gyrus versus ventromedial prefrontal lesions. Brain.

[76] Schilbach L., Timmermans B., Reddy V., Costall A., Bente G., Schlicht T., Vogeley K. (2013). Toward a second-person neuroscience. Behav. Brain Sci..

[77] Bolis D., Schilbach L. (2018). Observing and participating in social interactions: Action perception and action control across the autistic spectrum. Dev. Cogn. Neurosci..

[78] Hoehl S., Markova G. (2018). Moving developmental social neuroscience toward a second-person approach. PLoS Biol..

[79] Redcay E., Schilbach L. (2019). Using second-person neuroscience to elucidate the mechanisms of social interaction. Nat. Rev. Neurosci..

[80] Carollo A., Lim M., Aryadoust V., Esposito G. (2021). Interpersonal Synchrony in the Context of Caregiver-Child Interactions: A Document Co-citation Analysis. Front. Psychol..

[81] Hasson U., Ghazanfar A.A., Galantucci B., Garrod S., Keysers C. (2012). Brain-to-brain coupling: A mechanism for creating and sharing a social world. Trends Cogn. Sci..

[82] Jiang J., Dai B., Peng D., Zhu C., Liu L., Lu C. (2012). Neural synchronization during face-to-face communication. J. Neurosci..

[83] Liu N., Mok C., Witt E.E., Pradhan A.H., Chen J.E., Reiss A.L. (2016). NIRS-based hyperscanning reveals inter-brain neural synchronization during cooperative Jenga game with face-to-face communication. Front. Hum. Neurosci..

[84] Hoehl S., Fairhurst M., Schirmer A. (2021). Interactional synchrony: Signals, mechanisms and benefits. Soc. Cogn. Affect. Neurosci..

[85] Nguyen T., Schleihauf H., Kungl M., Kayhan E., Hoehl S., Vrtička P. (2020). Interpersonal Neural Synchrony During Father–Child Problem Solving: An fNIRS Hyperscanning Study. Child Dev..

[86] Nguyen T., Bánki A., Markova G., Hoehl S. (2020). Studying parent–child interaction with hyperscanning. Prog. Brain Res..

[87] Babiloni F., Astolfi L. (2014). Social neuroscience and hyperscanning techniques: Past, present and future. Neurosci. Biobehav. Rev..

[88] Nozawa T., Sasaki Y., Sakaki K., Yokoyama R., Kawashima R. (2016). Interpersonal frontopolar neural synchronization in group communication: An exploration toward fNIRS hyperscanning of natural interactions. Neuroimage.

[89] Balconi M., Vanutelli M.E. (2018). Functional EEG connectivity during competition. BMC Neurosci..

[90] Bevilacqua D., Davidesco I., Wan L., Chaloner K., Rowland J., Ding M., Poeppel D., Dikker S. (2019). Brain-to-brain synchrony and learning outcomes vary by student–teacher dynamics: Evidence from a real-world classroom electroencephalography study. J. Cogn. Neurosci..

[91] Cui X., Bryant D.M., Reiss A.L. (2012). NIRS-based hyperscanning reveals increased interpersonal coherence in superior frontal cortex during cooperation. Neuroimage.

[92] Fishburn F.A., Murty V.P., Hlutkowsky C.O., MacGillivray C.E., Bemis L.M., Murphy M.E., Huppert T.J., Perlman S.B. (2018). Putting our heads together: Interpersonal neural synchronization as a biological mechanism for shared intentionality. Soc. Cogn. Affect. Neurosci..

[93] Wass S.V., Noreika V., Georgieva S., Clackson K., Brightman L., Nutbrown R., Covarrubias L.S., Leong V. (2018). Parental neural responsivity to infants’ visual attention: How mature brains influence immature brains during social interaction. PLoS Biol..

[94] Cheng X., Li X., Hu Y. (2015). Synchronous brain activity during cooperative exchange depends on gender of partner: A fNIRS-based hyperscanning study. Hum. Brain Mapp..

[95] Baker J.M., Liu N., Cui X., Vrticka P., Saggar M., Hosseini S.H., Reiss A.L. (2016). Sex differences in neural and behavioral signatures of cooperation revealed by fNIRS hyperscanning. Sci. Rep..

[96] Pan Y., Cheng X., Zhang Z., Li X., Hu Y. (2017). Cooperation in lovers: An f NIRS-based hyperscanning study. Hum. Brain Mapp..

[97] Nguyen T., Schleihauf H., Kayhan E., Matthes D., Vrtička P., Hoehl S. (2020). The effects of interaction quality on neural synchrony during mother-child problem solving. Cortex.

[98] Miller J.G., Vrtička P., Cui X., Shrestha S., Hosseini S.H., Baker J.M., Reiss A.L. (2019). Inter-brain synchrony in mother-child dyads during cooperation: An fNIRS hyperscanning study. Neuropsychologia.

[99] Barker B., Iles J.E., Ramchandani P.G. (2017). Fathers, fathering and child psychopathology. Curr. Opin. Psychol..

[100] Azhari A., Leck W., Gabrieli G., Bizzego A., Rigo P., Setoh P., Bornstein M., Esposito G. (2019). Parenting stress undermines mother-child brain-to-brain synchrony: A hyperscanning study. Sci. Rep..

[101] Azhari A., Gabrieli G., Bizzego A., Bornstein M.H., Esposito G. (2023). Probing the association between maternal anxious attachment style and mother-child brain-to-brain coupling during passive co-viewing of visual stimuli. Attach. Hum. Dev..

[102] Azhari A., Lim M., Bizzego A., Gabrieli G., Bornstein M.H., Esposito G. (2020). Physical presence of spouse enhances brain-to-brain synchrony in co-parenting couples. Sci. Rep..

[103] Santamaria L., Noreika V., Georgieva S., Clackson K., Wass S., Leong V. (2020). Emotional valence modulates the topology of the parent-infant inter-brain network. NeuroImage.

[104] Reindl V., Gerloff C., Scharke W., Konrad K. (2018). Brain-to-brain synchrony in parent–child dyads and the relationship with emotion regulation revealed by fNIRS-based hyperscanning. NeuroImage.

[105] Quiñones-Camacho L.E., Fishburn F.A., Camacho M.C., Hlutkowsky C.O., Huppert T.J., Wakschlag L.S., Perlman S.B. (2020). Parent–child neural synchrony: A novel approach to elucidating dyadic correlates of preschool irritability. J. Child Psychol. Psychiatry.

[106] Fuemmeler B.F., Anderson C.B., Mâsse L.C. (2011). Parent-child relationship of directly measured physical activity. Int. J. Behav. Nutr. Phys. Act..

[107] Carter H.D. (1932). Family resemblances in verbal and numerical abilities. Genet. Psychololgy Monogr..

[108] DeFries J.C., Johnson R.C., Kuse A.R., McClearn G.E., Polovina J., Vandenberg S.G., Wilson J.R. (1979). Familial resemblance for specific cognitive abilities. Behav. Genet..

[109] Williams T. (1975). Family resemblance in abilities: The Wechsler scales. Behav. Genet..

[110] Willoughby R.R. (1927). Family similarities in mental-test abilities. Genet. Psychol. Monogr..

[111] Birch L.L. (1980). The relationship between children’s food preferences and those of their parents. J. Nutr. Educ..

[112] Rozin P., Fallon A., Mandell R. (1984). Family resemblance in attitudes to foods. Dev. Psychol..

[113] Field T. (1985). Attachment as psychobiological attunement: Being on the same wavelength. The Psychobiology of Attachment and Separation.

[114] Bornstein M.H., Putnick D.L., Cote L.R., Haynes O.M., Suwalsky J.T.D. (2015). Mother-infant contingent vocalizations in 11 countries. Psychol. Sci..

[115] Bornstein M.H., Putnick D.L., Park Y., Suwalsky J.T.D., Haynes O.M. (2017). Human infancy and parenting in global perspective: Specificity. Proc. R. Soc. B.

[116] Bornstein M.H. (2022). Parenting, Infancy, Culture: Specificity and Commonality in Argentina, Belgium, Israel, Italy, and the United States.

[117] Field T., Healy B., Goldstein S., Guthertz M. (1990). Behavior-state matching and synchrony in mother-infant interactions of nondepressed versus depressed dyads. Dev. Psychol..

[118] Beeghly M., Perry B.W., Cicchetti D. (1989). Structural and affective dimensions of play development in young children with Down Syndrome. Int. J. Behav. Dev..

[119] Bornstein M.H., Putnick D.L., Suwalsky J.T.D., Venuti P., de Falco S., Zingman de Galperín C., Gini M., Heslington Tichovolsky M. (2012). Emotional relationships in mothers and infants: Culture-common and community-specific characteristics of dyads from rural and metropolitan settings in Argentina, Italy, and the United States. J. Cross-Cult. Psychol..

[120] Crawley S.B., Spiker D. (1983). Mother-child interactions involving two-year-olds with Down Syndrome: A look at individual differences. Child Dev..

[121] Collins W.A., Maccoby E.E., Steinberg L., Hetherington E.M., Bornstein M.H. (2000). Contemporary research on parenting: The case for nature and nurture. Am. Psychol..

[122] Maccoby E.E. (1992). The role of parents in the socialization of children: An historical overview. Dev. Psychol..

[123] Ainsworth M.D. (1989). Attachments beyond infancy. Am. Psychol..

[124] Feldman R., Eidelman A.I. (2004). Parent–infant synchrony and the social–emotional development of triplets. Dev. Psychol..

[125] Sander L. (2000). Where are we going in the field of infant mental health?. Infant Ment. Health J..

[126] Sroufe L.A., Egeland B., Carlson E.A., Collins W.A. (2005). The Development of the Person: The Minnesota Study of Risk and Adaptation from Birth to Adulthood.

